# Role of gender in short- and long-term outcomes after surgery for type A aortic dissection: analysis of a multicentre European registry

**DOI:** 10.1093/ejcts/ezae242

**Published:** 2024-06-26

**Authors:** Francesco Onorati, Alessandra Francica, Till Demal, Francesco Nappi, Sven Peterss, Joscha Buech, Antonio Fiore, Thierry Folliguet, Andrea Perrotti, Amélie Hervé, Lenard Conradi, Angelo M Dell’Aquila, Andreas Rukosujew, Angel G Pinto, Javier Rodriguez Lega, Marek Pol, Jan Rocek, Petr Kacer, Konrad Wisniewski, Enzo Mazzaro, Igor Vendramin, Daniela Piani, Luisa Ferrante, Mauro Rinaldi, Eduard Quintana, Robert Pruna-Guillen, Sebastien Gerelli, Metesh Acharya, Giovanni Mariscalco, Mark Field, Manoj Kuduvalli, Matteo Pettinari, Stefano Rosato, Paola D’Errigo, Mikko Jormalainen, Caius Mustonen, Timo Mäkikallio, Dario Di Perna, Tatu Juvonen, Giuseppe Gatti, Giovanni Battista Luciani, Fausto Biancari

**Affiliations:** Division of Cardiac Surgery, University of Verona Medical School, Verona, Italy; Division of Cardiac Surgery, University of Verona Medical School, Verona, Italy; Department of Cardiovascular Surgery, University Heart and Vascular Center Hamburg, Hamburg, Germany; Department of Cardiac Surgery, Centre Cardiologique du Nord de Saint-Denis, Paris, France; Department of Cardiac Surgery, LMU University Hospital, Ludwig Maximilian University, Munich, Germany; Department of Cardiac Surgery, LMU University Hospital, Ludwig Maximilian University, Munich, Germany; German Centre for Cardiovascular Research, Partner Site Munich Heart Alliance, Munich, Germany; Department of Cardiac Surgery, Hôpitaux Universitaires Henri Mondor, Assistance Publique-Hôpitaux de Paris, Creteil, France; Department of Cardiac Surgery, Hôpitaux Universitaires Henri Mondor, Assistance Publique-Hôpitaux de Paris, Creteil, France; Department of Thoracic and Cardiovascular Surgery, University of Franche-Comte, Besancon, France; Department of Thoracic and Cardiovascular Surgery, University of Franche-Comte, Besancon, France; Department of Cardiovascular Surgery, University Heart and Vascular Center Hamburg, Hamburg, Germany; Department of Cardiothoracic Surgery, University Hospital Muenster, Muenster, Germany; Department of Cardiothoracic Surgery, University Hospital Muenster, Muenster, Germany; Cardiovascular Surgery Department, University Hospital Gregorio Marañón, Madrid, Spain; Cardiovascular Surgery Department, University Hospital Gregorio Marañón, Madrid, Spain; Department of Cardiac Surgery, Third Faculty of Medicine, Charles University and University Hospital Kralovske Vinohrady, Prague, Czech Republic; Department of Cardiac Surgery, Third Faculty of Medicine, Charles University and University Hospital Kralovske Vinohrady, Prague, Czech Republic; Department of Cardiac Surgery, Third Faculty of Medicine, Charles University and University Hospital Kralovske Vinohrady, Prague, Czech Republic; Department of Cardiothoracic Surgery, University Hospital Muenster, Muenster, Germany; Division of Cardiac Surgery, Cardio-Thoracic and Vascular Department, Azienda Sanitaria Universitaria Giuliano Isontina, Trieste, Italy; Cardiothoracic Department, University Hospital, Udine, Italy; Cardiothoracic Department, University Hospital, Udine, Italy; Cardiac Surgery, Molinette Hospital, University of Turin, Turin, Italy; Cardiac Surgery, Molinette Hospital, University of Turin, Turin, Italy; Department of Cardiovascular Surgery, Hospital Clínic de Barcelona, University of Barcelona, Barcelona, Spain; Department of Cardiovascular Surgery, Hospital Clínic de Barcelona, University of Barcelona, Barcelona, Spain; Department of Cardiac Surgery, Centre Hospitalier Annecy Genevois, Epagny Metz-Tessy, France; Department of Cardiac Surgery, Glenfield Hospital, Leicester, UK; Department of Cardiac Surgery, Glenfield Hospital, Leicester, UK; Liverpool Centre for Cardiovascular Sciences, Liverpool Heart and Chest Hospital, Liverpool, UK; Liverpool Centre for Cardiovascular Sciences, Liverpool Heart and Chest Hospital, Liverpool, UK; Department of Cardiac Surgery, Ziekenhuis Oost Limburg, Genk, Belgium; National Center for Global Health, Istituto Superiore di Sanitá, Rome, Italy; National Center for Global Health, Istituto Superiore di Sanitá, Rome, Italy; Heart and Lung Center, Helsinki University Hospital, University of Helsinki, Helsinki, Finland; Heart and Lung Center, Helsinki University Hospital, University of Helsinki, Helsinki, Finland; Department of Medicine, South-Karelia Central Hospital, University of Helsinki, Lappeenranta, Finland; Department of Cardiac Surgery, Centre Hospitalier Annecy Genevois, Epagny Metz-Tessy, France; Heart and Lung Center, Helsinki University Hospital, University of Helsinki, Helsinki, Finland; Research Unit of Surgery, Anesthesia and Critical Care, University of Oulu, Oulu, Finland; Division of Cardiac Surgery, Cardio-Thoracic and Vascular Department, Azienda Sanitaria Universitaria Giuliano Isontina, Trieste, Italy; Division of Cardiac Surgery, University of Verona Medical School, Verona, Italy; Heart and Lung Center, Helsinki University Hospital, University of Helsinki, Helsinki, Finland; Department of Medicine, South-Karelia Central Hospital, University of Helsinki, Lappeenranta, Finland

**Keywords:** Type A aortic dissection, Gender, Sex, Acute aortic syndrome

## Abstract

**OBJECTIVES:**

Gender difference in the outcome after type A aortic dissection (TAAD) surgery remains an issue of ongoing debate. In this study, we aimed to evaluate the impact of gender on the short- and long-term outcome after surgery for TAAD.

**METHODS:**

A multicentre European registry retrospectively included all consecutive TAAD surgery patients between 2005 and 2021 from 18 hospitals across 8 European countries. Early and late mortality, and cumulative incidence of aortic reoperation were compared between genders.

**RESULTS:**

A total of 3902 patients underwent TAAD surgery, with 1185 (30.4%) being females. After propensity score matching, 766 pairs of males and females were compared. No statistical differences were detected in the early postoperative outcome between genders. Ten-year survival was comparable between genders (47.8% vs 47.1%; log-rank test, *P* = 0.679), as well as cumulative incidences of distal or proximal aortic reoperations. Ten-year relative survival compared to country-, year-, age- and sex-matched general population was higher among males (0.65) compared to females (0.58). The time-period subanalysis revealed advancements in surgical techniques in both genders over the years. However, an increase in stroke was observed over time for both populations, particularly among females.

**CONCLUSIONS:**

The past 16 years have witnessed marked advancements in surgical techniques for TAAD in both males and females, achieving comparable early and late mortality rates. Despite these findings, late relative survival was still in favour of males.

## Graphical Abstract

**Figure:**
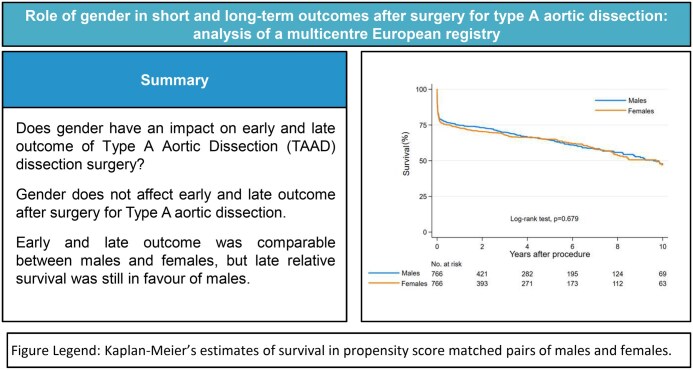

## INTRODUCTION

Recently, the gender gap is gaining great interest because of the evidence that management of women with cardiovascular disease is mostly based on studies including only or mainly men [1, 2]. Type A aortic dissection (TAAD) remains a highly lethal cardiovascular emergency requiring immediate surgery, and despite the improvement in surgical techniques and perioperative care, the mortality is still high [3–5]. Even though some studies have investigated the differences between males and females in TAAD surgery, data are still scarce, often with contradictory results [4–9]. Moreover, data about the impact of gender on long-term outcome have rarely been reported, and efforts to increase the body of knowledge on this topic have recently been advocated [10, 11]. The present multicentre study aimed to explore the differences in surgical management of TAAD in males and females in the last 16 years, and to evaluate the impact of gender on short- to long-term outcome after surgery for acute TAAD.

## PATIENTS AND METHODS

### Ethical statement

The study protocol was approved by the Ethical Review Board of the Helsinki University Hospital, Finland (21 April 2021, diary no. HUS/237/2021) and the Ethical Review Board of each participating hospital. Informed consent was waived due to the retrospective nature of the registry. The trial is registered on ClinicalTrials.gov (Identifier: NCT04831073).

### Study design and population

The European Registry of Type A Aortic Dissection is a multicentre, retrospective cohort study. All consecutive TAAD patients operated at 18 centres of cardiac surgery located in 8 European countries (Belgium, Czech Republic, Finland, France, Germany, Italy, Spain and the UK) from 1 January 2005 to 31 March 2021 were included in the registry [12]. A Microsoft Access datasheet (Redmond, Washington, USA) was used for collection of data on prespecified baseline, operative and outcome variables. This study followed the Strengthening the Reporting of Observational Studies in Epidemiology (STROBE) guidelines [13]. Data on the date of death and repeated aortic intervention were collected retrospectively from national electronic registries as well as by contacting regional hospitals, patients and their relatives. The follow-up ended on 31 December 2021. The inclusion criteria of the European Registry of Type A Aortic Dissection registry were as follows: patients aged >18 years, TAAD or type A intramural haematoma involving the ascending aorta, primary surgical repair of acute TAAD and symptoms started within 7 days prior to surgery. Patients with any concomitant procedures were included. The exclusion criteria were the following: patients aged < 18 years, onset of symptoms >7 days prior to surgery, prior procedure for TAAD, type B dissection (with primary tear located in the descending aorta with retrograde progression), concomitant endocarditis, TAAD secondary to blunt or penetrating chest trauma and pregnancy status.

### Objectives

The primary objectives of this study were 10-year survival and cumulative incidence of proximal and distal aortic reoperations. Secondary objectives were the following: in-hospital mortality, defined as all-cause death occurred during the index hospitalization, any neurological complications, stroke, paraplegia, tetraplegia, need of mechanical circulatory support, heart failure, temporary or permanent dialysis, sepsis, laryngeal nerve palsy, reoperation because of intrathoracic bleeding, deep sternal wound infection, tracheostomy, mesenteric and peripheral ischaemia, additional procedures for ischaemic complications and length of stay in the intensive care unit. Definition criteria of these outcome measures have been previously reported [11].

Finally, a subanalysis based on time periods was conducted, comparing 3 study periods (2005–2009, 2010–2014 and 2015–2021) regarding surgical techniques and postoperative outcomes in both males and females.

The data underlying this article are available in the article and in its online supplementary material.

### Statistical analysis

Continuous variables were reported as means and standard deviations as well as median and interquartile range (IQR) when appropriate. Categorical variables were reported as counts and percentages. Shapiro–Wilk test was used to assess the distribution of quantitative variables. The chi-squared test and the Fisher’s exact test were used to analyse groups differences between categorical variables, and the Student’s *t*-test was used to compare continuous variables. To account for imbalances between cohorts, a propensity score was calculated by logistic regression considering the statistically significant differences among baseline variables between the 2 study cohorts (age, hypertension, pulmonary disease, renal malperfusion, peripheral malperfusion, preoperative arterial lactate, iatrogenic TAAD, moderate-to-severe frailty). Males and females were matched using the nearest neighbour method using a caliper width of 0.2 of the standard deviation of the logit. Standardized difference <0.1 was considered a non-significant imbalance between the covariates of the matched study cohorts. Survival rate was estimated with the Kaplan–Meier method and compared using the log-rank test with a without stratification for 5 propensity score strata. Person-time and the incidence rates of mortality were estimated. Smoothed hazard estimates of mortality of the study cohorts were estimated and plotted. Cox proportional hazards analysis was used to adjust the study cohorts for major confounders. Cox proportional hazards analysis was used also to regress survival on gender and a categorical variable denoting the 5 propensity score strata. Complications requiring aortic reoperations might be hindered by death of the patient occurring during follow-up. Therefore, competing risk analysis using the Fine-Gray test with all-cause death as a competing event was performed to estimate the hazard and cumulative incidence of distal and proximal aortic reoperations. Risk estimates are reported as odds ratios, hazard ratios (HR) and subdistributional hazard ratios (SHRs) with their 95% confidence intervals (CIs). A time-period subanalysis was performed considering 3 different study periods (2005–2009 vs 2010–2014 vs 2015–2021). Three different propensity score matching analyses were performed for each of these study periods to account for baseline differences of the 2 study cohorts at each timeframe. Surgical management and postoperative outcomes were then compared between males and females for each period and across time. Finally, a logistic regression model was used to perform a multivariable analysis to identify independent predictors of stroke among preoperative and intraoperative variables for each gender. A *P* < 0.05 in univariate analysis was used to include variables into multivariable regression models. Country-, year-, age- and sex-matched cumulative expected survival and cumulative relative survival were estimated using the Ederer II method with the *strs* module for Stata. Relative survival is the ratio of the proportion of observed survivors in a cohort of patients to the proportion of expected survivors in a comparable set of disease-free subjects. A relative survival <1.0 indicates that the observed survival of patients is lower than the expected survival of subjects from the general population. Mortality rates of the general population of each participating country were retrieved from the Human Mortality Database (https://www.mortality.org/). Statistical analyses were performed with the SPSS (version 29.0, SPSS Inc., IBM, Chicago, Illinois, USA) and Stata (version 15.1, StataCorp LLC, College Station, Texas, USA) statistical software.

## RESULTS

### Overall population

From 2005 to 2021, 3902 consecutive patients underwent surgery for acute TAAD across 18 European Cardiac Surgery centres. Among them, 2717 (69.6%) were males, and 1185 (30.4%) were females. Notably, females were significantly older (68.1 ± 12.2 vs 61.2 ± 12.8 years; *P* < 0.001) and exhibited higher frailty, along with a higher incidence of hypertension and chronic lung disease. Upon hospital admission, renal and peripheral malperfusion were more frequently diagnosed in males, leading to higher arterial lactate levels. A comprehensive summary of all baseline characteristics is provided in Supplementary Material, Table S1.

Intraoperatively, males presented a higher incidence of bicuspid aortic valve and more extensive aortic dissection, characterized by a higher incidence of tears in the aortic root or arch, with more frequent involvement of the right coronary and left coronary sinus compared to females (Supplementary Material, Table S2).

Right axillary artery cannulation was the preferred method of cardiopulmonary bypass (CPB) in male patients, and complex aortic root repair or total arch replacement was predominantly performed in males compared to females (Supplementary Material, Table S2). Antegrade cerebral perfusion during hypothermic arrest was equally employed for cerebral protection in both groups. The duration of CPB was longer in male patients (Supplementary Material, Table S2). No significant differences in in-hospital mortality (17.7% vs 17.6%; *P* = 0.982) or neurological complications were observed between genders (Supplementary Material, Table S3). However, males exhibited a higher incidence of sepsis, acute kidney injury requiring dialysis, lower limb ischaemia and surgery for mesenteric ischaemia (Supplementary Material, Table S3).

The mean follow-up time of the entire series was 3.9 ± 4.2 years (median, 2.5 years, IQR 6.3), that of males was 4.1 ± 4.2 years (median, 2.7 years, IQR 6.5) and of females was 3.6 ± 4.0 years (median, 2.2 years, IQR 5.8). Females had lower crude 10-year survival compared to males (54.5% vs 46.6%; *P* = 0.018) (Supplementary Material, Fig. S1). Crude cumulative incidences of proximal (males 4.8% vs females 3.3%, SHR 0.682, 95% CI 0.433–1.074; *P* = 0.098) and distal (males 8.0% vs females 6.4%; SHR 0.812, 95% CI 0.587–1.122; *P* = 0.207) aortic reoperations were comparable between the study cohorts (Supplementary Material, Fig. S2A and B).

### Propensity score matching analysis

Propensity score matching yielded 766 pairs of males and females with similar preoperative risk profiles (Table 1). The overlaying graphs of males and females distribution before and after propensity score matching are shown in Supplementary Material, Fig. S3. Aortic root replacement was more frequently performed in males (31.9% vs 19.3%; *P* < 0.001), even though the incidence of tear of the aortic root was comparable between genders. Tears in the aortic arch remained more frequent in male patients, as well as the proportion of total aortic arch repair (16.5% vs 10.3%; *P* < 0.001). However, in more than 50% of males and females who underwent total aortic arch repair, any tear in the arch was not detected (*P* = 0.78) (Supplementary Material, Table S4). Consonant with these observations, myocardial ischaemia time and CPB duration were longer in males (Table 2). No differences between genders were observed in terms of in-hospital mortality and postoperative complications, except for a higher rate of temporary dialysis in males (Table 3).

**Table 1:** Propensity score-matched cohorts: baseline patient characteristics

Variables	Males No. 766	Females No. 766	SD
Age, years	67.1 (11.9)	67 (11.9)	0.009
Genetic syndromes	10 (1.3)	20 (2.6)	0.094
Marfan syndrome	10 (1.3)	19 (2.6)	0.086
Loeys–Dietz syndrome	0	0	–
Ehlers–Danlos syndrome	0	1 (0.1)	0.040
Family history of aortic aneurysm	52 (6.8)	50 (6.5)	0.010
Family history of aortic dissection	21 (2.7)	25 (3.3)	0.031
Aortitis	4 (0.5)	4 (0.5)	0.000
Prior cardiac surgery	27 (3.5)	26 (3.4)	0.005
Iatrogenic TAAD	21 (2.7)	21 (2.7)	0.000
Hypertension	580 (75.8)	570 (74.4)	0.030
Diabetes	35 (4.6)	46 (6.0)	0.064
Prior stroke	41 (5.4)	30 (3.9)	0.068
Pulmonary disease	79 (10.3)	76 (9.9)	0.013
Extracardiac arteriopathy	47 (6.1)	54 (7.0)	0.037
Moderate-to-severe frailty (CFS grades 6–9)	6 (0.8)	14 (1.8)	0.092
Exposure to antiplatelet drugs^a^	59 (7.7)	31 (4)	0.156
Exposure to oral anticoagulant	23 (3.0)	26 (3.4)	0.028
Penn classification			0.071
a	425 (55.5)	417 (49.5)	
b	201 (2.7)	189 (2.5)	
c	53 (6.9)	57 (7.4)	
b + c	87 (11.4)	103 (13.4)	
Cardiac massage	31 (4)	31 (4.0)	0.000
Invasive mechanical ventilation	72 (9.4)	84 (11.7)	0.052
Acute renal failure	24 (3.1)	30 (3.9)	0.042
Cardiogenic shock requiring inotropes	135 (17.6)	153 (20)	0.060
Cerebral malperfusion	163 (21.3)	192 (25.1)	0.090
Spinal malperfusion	14 (1.8)	12 (1.6)	0.020
Renal malperfusion	75 (9.8)	69 (9.0)	0.037
Mesenteric malperfusion	31 (4)	29 (3.8)	0.013
Peripheral malperfusion	105 (13.7)	106 (13.8)	0.004
Arterial lactate, mmol/l	2.3 (2.1)	2.3 (2.2)	0.001

Continuous variables are reported as mean and standard deviation (in parentheses). Categorical variables are reported as counts and percentages (in parentheses).

a Clopidogrel, ticagrelor, ticlopidine, prasugrel.

CFS: clinical frailty scale; SD: standardized differences; TAAD: type A aortic dissection.

**Table 2:** Propensity score-matched cohorts: intraoperative findings and operative data

Variables	Males No. 766	Females No. 766	*P*-value
Intraoperative findings			
Site of aortic dissection tear			
Aortic root	111 (14.5)	111 (14.5)	1.00
Ascending aorta	533 (69.6)	548 (71.8)	0.40
Aortic arch	140 (18.3)	107 (14.0)	0.02
Dissection involving sinuses of Valsalva			
Non-coronary sinus	162 (21.1)	150 (19.6)	0.45
Right coronary sinus	137 (17.9)	139 (18.1)	0.89
Left coronary sinus	73 (9.5)	67 (47.9)	0.60
Bicuspid aortic valve	25 (3.3)	16 (2.1)	0.15
Operative data			
Arterial cannulation site			
Ascending/aortic arch	104 (13.6)	143 (18.7)	0.007
Innominate artery	92 (12)	70 (9.1)	0.07
Right subclavian/axillary artery	354 (46.2)	323 (42.2)	0.11
Common femoral artery	227 (14.8)	235 (30.7)	0.66
Proximal aortic repair			
Supracoronary aortic replacement	558 (72.8)	607 (79.2)	0.003
Aortic root replacement	208 (27.2)	159 (20.8)	0.003
Distal ascending aorta anastomosis	217 (28.3)	251 (32.8)	0.06
Aortic valve replacement	45 (5.9)	40 (5.2)	0.57
Aortic arch repair			
Hemiarch repair	368 (48)	370 (48.3)	0.91
Total arch repair	112 (14.6)	81 (10.6)	0.017
Frozen elephant trunk procedure	43 (5.6)	35 (4.6)	0.35
Conventional elephant trunk procedure	30 (3.9)	16 (2.1)	0.04
TEVAR during the index hospitalization	4 (0.5)	3 (0.4)	0.70
Cerebral perfusion strategy			
Antegrade	525 (68.5)	486 (63.4)	0.03
Retrograde	68 (8.9)	64 (8.4)	0.71
Myocardial ischaemic time, min	125 (57)	111 (55)	<0.001
CPB time, min	223 (89)	201 (86)	<0.001

Continuous variables are reported as mean and standard deviation (in parentheses). Categorical variables are reported as counts and percentages (in parentheses).

CPB: cardiopulmonary bypass time; TEVAR: thoracic endovascular aortic repair.

**Table 3:** Propensity score-matched cohorts: early postoperative outcomes

Outcomes	Males No. 766	Females No. 766	*P*-value
In-hospital death	143 (18.7)	146 (19.1)	0.84
Any neurological complication	148 (19.3)	175 (22.8)	0.09
Stroke	128 (16.7)	154 (20.1)	0.09
Paraplegia/paraparesis	31 (4.0)	46 (6.0)	0.08
Tetraplegia	0	0	**–**
Sepsis	109 (14.2)	97 (12.7)	0.37
Dialysis	132 (17.2)	100 (13.1)	0.02
Laryngeal nerve palsy	11 (1.4)	12 (1.6)	0.83
Reoperation for intrathoracic bleeding	103 (13.4)	89 (11.6)	0.28
Deep sternal wound infection	26 (3.4)	33 (4.3)	0.35
Tracheostomy	59 (7.7)	63 (8.2)	0.70
Heart failure	122 (15.9)	113 (14.8)	0.52
IABP support	6 (0.8)	10 (1.3)	0.31
ECMO support	14 (1.8)	18 (2.3)	0.47
Mesenteric ischaemia	26 (3.4)	33 (4.3)	0.35
Acute upper limb ischaemia	2 (0.3)	3 (0.4)	1.0
Acute lower limb ischaemia	25 (3.3)	17 (2.2)	0.21
Additional procedures for complications			
Major lower limb amputation	2 (0.3)	1 (0.1)	1.0
Revascularization procedure for upper limb ischaemia	2 (0.3)	2 (0.3)	1.0
Revascularization procedure for lower limb ischaemia	10 (1.3)	9 (1.2)	0.82
Revascularization for mesenteric ischaemia	3 (0.4)	1 (0.1)	0.63
Revascularization for renal ischaemia	2 (0.3)	0	0.50
Surgery for intestinal complication	4 (0.5)	1 (0.1)	0.37
Aortic fenestration	2 (0.3)	0	0.16
ICU stay, days	9.8 (13.2)	9.1 (10.9)	0.19

Continuous variables are reported as mean and standard deviation (in parentheses). Categorical variables are reported as counts and percentages (in parentheses).

ECMO: extracorporeal membrane oxygenation; IABP: intra-aortic balloon pump; ICU: intensive care unit.

At 10-year, person-time was 2675 patient/years for males and 2509 patient/years for females, and the incidence rates of mortality were 0.11 (95% CI 0.10–0.11) and 0.10 (95% CI 0.09–0.12), respectively.

**Figure 1:**
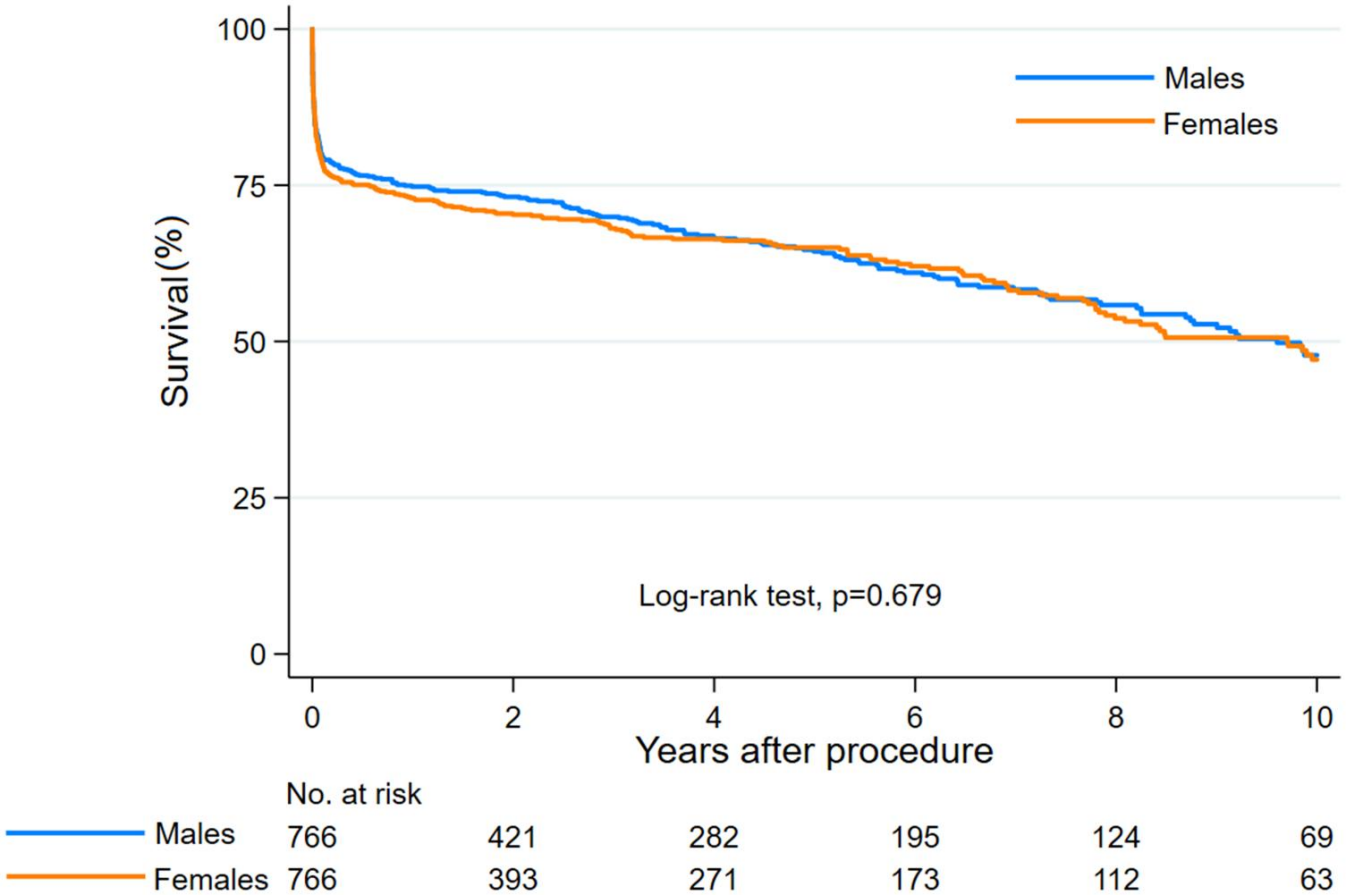
Kaplan–Meier estimates of survival in propensity score-matched pairs of males and females.

Males and females had comparable 10-year survival (47.8% vs 47.1%; log-rank test, *P* = 0.679; log-rank test stratified for 5 propensity score strata, *P* = 0.756; HR 1.035, 95% CI 0.878–1.222; Cox regression stratified for 5 propensity score strata, HR 1.030, 95% CI 0.872–1.215) (Fig. 1). Smoothed hazard estimates of mortality showed a biphasic trend of mortality after surgery which was lower among females compared to males between 2 and 5 years after surgery which was inverted later (Supplementary Material, Fig. S4).

**Figure 2:**
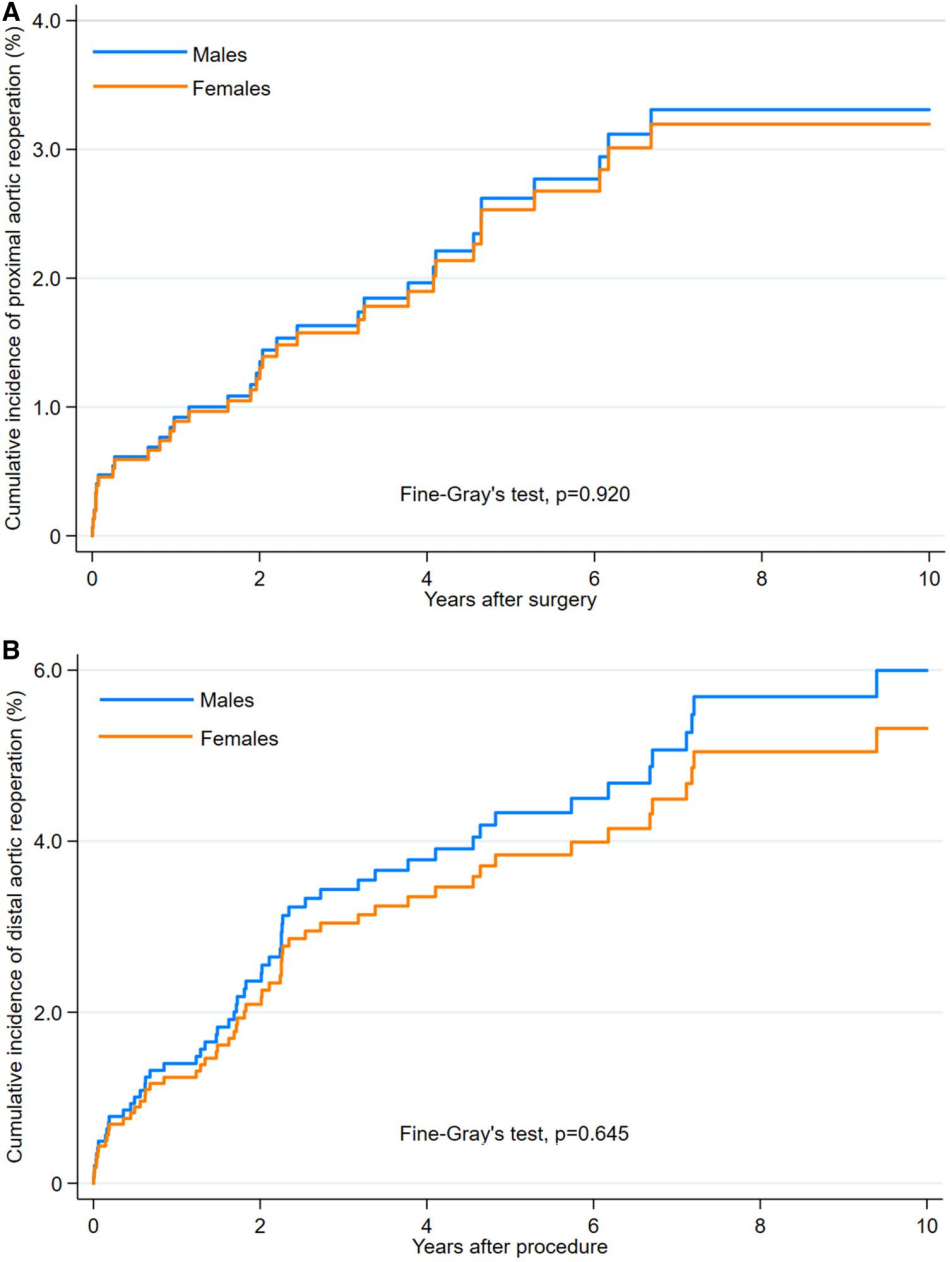
Cumulative incidences of proximal (**A**) and distal (**B**) aortic reoperations in the propensity score-matched pairs of males and females.

Cox regression adjusted for time period showed no differences between the study cohorts in terms of 10-year survival (HR 1.040, 95% CI 0.881–1.227; *P* = 0.643). Cumulative incidences of proximal (males 3.2% vs females 3.3%, SHR 0.966, 95% CI 0.488–1.910; *P* = 0.920) and distal (males 6.0% vs females 5.3%; SHR 0.883, 95% CI 0.523–1.493; *P* = 0.645) aortic reoperations were comparable between the study cohorts (Fig. 2A and B).

Ten-year expected survival of country-, year-, age- and sex-matched general population was 72.1% for males and 80.8% for females. Ten-year relative survival was higher for males (0.65) compared to females (0.58) (Supplementary Material, Fig. S5).

### Time-period subanalysis

**Figure 3:**
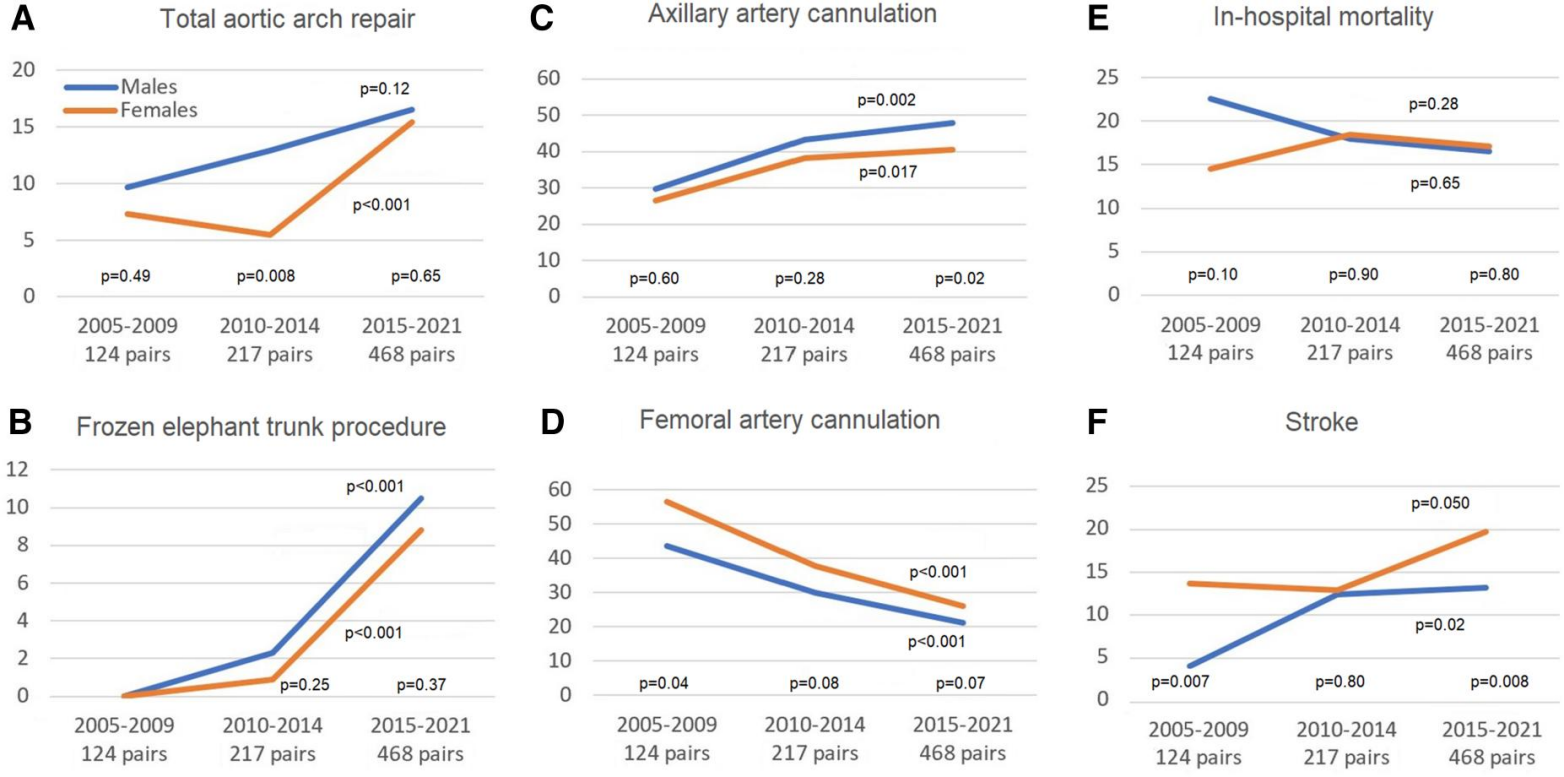
Time-period subanalysis: temporal trends in total aortic arch replacement (**A**), frozen elephant trunk procedure (**B**), peripheral arterial cannulation (**C, D**), in-hospital mortality (**E**) and stroke (**F**).

After 3 different propensity score matching analyses, 124 pairs, 217 pairs and 468 pairs of males and females were yielded for the study periods 2005–2009, 2010–2014 and 2015–2021, respectively (Supplementary Material, Tables S5–S7). Surgical management and postoperative complications were explored and compared between genders for each period and throughout time. The analysis revealed that antegrade perfusion has become the preferred cerebral protection strategy across the years (*P* < 0.001), reaching 70% in both males and females (2015–2021; *P* = 0.83). Complex arch surgery has also become more prevalent over time in both genders. Specifically, the frozen elephant trunk technique started to be used 10 years ago, and its use rapidly increased in both genders through the last years (Fig. 3A and B, Supplementary Material, Table S8). The use of the axillary artery cannulation has increased over time in both genders, although it remained less frequent in females (2015–2021: 47.9% vs 40.6%; *P* = 0.008). On the contrary, femoral artery cannulation showed a declining trend over time in both groups. Nevertheless, femoral artery cannulation remains more prevalent among females (Fig. 3C and D, Supplementary Material, Table S8). Aortic root replacement was less frequently performed over the last decade in both genders, but it remained more frequently used in males (Supplementary Material, Table S8). In-hospital mortality and major postoperative complications were comparable between genders across the 3 time periods, with the only exception of postoperative stroke. The incidence of stroke significantly increased over time in both study cohorts, although it remained higher in females, particularly during the last 5 years (Fig. 3E and F, Table 4).

**Table 4:** Time-period subanalysis: postoperative outcome in males and females in different study periods

	2005–2009 124 pairs	2010–2014 217 pairs	2015–2021 468 pairs	
In-hospital mortality				*P*-value
Males, *n* (%)	28 (22.6)	39 (18)	77 (16.5)	*0.28*
Females, *n* (%)	18 (14.5)	40 (18.4)	80 (17.1)	*0.65*
*P*-value	*0.10*	*0.90*	*0.80*	
Stroke				
Males, *n* (%)	5 (4.0)	27 (12.4)	62 (13.2)	*0.02*
Females, *n* (%)	17 (13.7)	28 (12.9)	92 (19.7)	*0.05*
*P*-value	*0.007*	*0.8*	*0.008*	
Paraplegia				
Males, *n* (%)	2 (1.6)	8 (3.7)	7 (1.5)	*0.16*
Females, *n* (%)	7 (5.6)	6 (2.8)	10 (2.1)	*0.11*
*P*-value	0.90	0.60	0.46	
Sepsis				
Males, *n* (%)	17 (13.7)	27 (12.4)	48 (10.3)	*0.47*
Females, *n* (%)	10(8.1)	16 (7.4)	57 (12.2)	*0.11*
*P*-value	*0.15*	*0.07*	*0.35*	
Reoperation for bleeding				
Males, *n* (%)	23 (18.5)	27 (12.4)	72 (15.4)	*0.30*
Females, *n* (%)	15 (12.1)	26 (12)	50 (10.7)	*0.83*
*P*-value	*0.16*	*0.88*	*0.033*	
Dialysis				
Males, *n* (%)	24 (19.4)	25 (11.5)	48 (10.3)	*0.02*
Females, *n* (%)	13 (10.5)	23 (10.6)	58 (12.4)	*0.71*
*P*-value	*0.05*	*0.76*	*0.29*	
Tracheostomy				
Males, *n* (%)	10 (8.1)	20 (9.2)	35 (7.5)	*0.74*
Females, *n* (%)	12 (9.7)	17 (3.9)	36 (7.7)	*0.76*
*P*-value	*0.65*	*0.61*	*0.90*	
Mechanical circulatory support				
Males, *n* (%)	4 (3.2)	7 (3.2)	18 (3.8)	*0.89*
Females, *n* (%)	5 (4)	7 (3.2)	17 (3.6)	*0.92*
*P*-value	*0.73*	*1.0*	*0.86*	
Mesenteric ischaemia				
Males, *n* (%)	4 (3.2)	7 (3.2)	11 (2.4)	*0.75*
Females, *n* (%)	5 (4.0)	4 (1.8)	19 (4.1)	*0.31*
*P*-value	*1.0*	*0.36*	*0.14*	
Lower limb ischaemia				
Males, *n* (%)	3 (2.4)	5 (2.3)	13 (2.8)	*0.93*
Females, *n* (%)	1 (0.8)	4 (1.8)	11 (2.4)	*0.54*
*P*-value	*0.60*	*0.74*	*0.68*	

Finally, preoperative cerebral malperfusion, exposure to antiplatelet drugs, history of stroke, frozen elephant trunk procedure and prolonged CPB time emerged as independent predictors of stroke in males (Supplementary Material, Table S9). Conversely, cerebral malperfusion, preoperative mechanical ventilation and elevated arterial lactate levels at admission were identified as independent risk factors of stroke among females. Noteworthy, no surgical or technical variables were found to be predictive of stroke among females (Supplementary Material, Table S10).

## DISCUSSION

This study is a large-scale European multicentre investigation reporting gender-related clinical presentation, surgical management and outcome of TAAD surgery at short- to long-term follow-up.

Females represented one-third of the overall population and were significantly older, consistent with data reported by the other international registries [5, 8, 14, 15]. Indeed, in the latest report from the International Registry of Acute Aortic Dissection (IRAD) [5, 8], the German Registry for Acute Aortic Dissection Type A (GERAADA) [14] and the Nordic Consortium for Acute Type A Aortic Dissection (NORCAAD) [15] registries, female prevalence ranged from 30% to 37%, with a mean age over 65 years, while males were younger, with a mean age of 60 years. Advanced age at onset of TAAD aligns with the incidence of cardiovascular disease, attributed to the protective effect of oestrogen [16]. Of note, oestrogen reduces the stiffness of the aortic and other arteries wall [17], suggesting a potential role in preventing aortic dissection until menopause.

Additionally, females more often had a history of hypertension and chronic pulmonary disease, consistent with the NORCAAD [15] and the IRAD [5, 8] reports, whereas male patients were more commonly affected by peripheral and renal malperfusion, according to the German registry [14]. The higher prevalence of hypertension and chronic pulmonary disease in females may be explained by higher age at presentation, as comorbidities trend with age. On the other hand, male patients of our cohort exhibited a more extensive aortic disease, characterized by a higher incidence of tears in the aortic root and arch, as well as involvement of visceral and peripheral arteries, explaining peripheral and renal malperfusion at referral. While these findings contradict some previous studies reporting a more aggressive disease in female patients [6, 18], our results align with the latest GERAADA [14] and IRAD reports [8]. Similar results were also recently demonstrated in a meta-analysis by Meccanici *et al.* [19], which reported a more proximally located dissections in females compared to males.

Our study also revealed some differences in the surgical approach. In both unmatched and matched cohorts, male patients underwent more extensive aortic root and aortic arch replacement, irrespective of the presence of intimal tear. These data possibly explain the longer CPB time in males compared to females, who appeared to undergo a more conservative approach. This trend is consistent with 2 recent meta-analyses: Meccanici *et al.* [19], analysing 9 studies from 1999 to 2020, and Carbone *et al.* [20], analysing 16 studies from 2004 to 2022, found that females received less complex surgical repairs than men, resulting in shorter operative times. Similar findings were also reported by Huckaby *et al.* [14] in the latest IRAD report. Conversely, the German and the Nordic registries [8, 15] indicated that different aortic arch repair strategies were similarly distributed between genders. However, aortic root replacement was more frequently used in males than females also in these registries. Overall, these findings suggest a trend towards a less complex surgical approach to female patients with TAAD.

Nonetheless, despite different preoperative risk profiles and surgical approaches, in-hospital mortality was similar between genders. This trend was also confirmed in the matched population, with a reported 17% in-hospital mortality. This finding contradicts previous studies, which reported higher overall mortality and worse outcomes in females. The first IRAD on this issue reported a poorer early surgical outcome in females compared to males (32% vs 22%), indicating a higher in-hospital mortality despite a similar time-delay, similar surgical techniques and haemodynamic status at admission [5]. Similarly, a Swedish study reported a higher 30-day mortality in females (26% vs 21%; *P* < 0.001) [4]. However, these results might reflect the results of surgery from the past decades, which did not account for the recent advancements in surgery and perioperative care. In line with that, Huckaby *et al.* [14] observed a decreased mortality from 33% to 22% over the last 2 decades. Furthermore, even though the authors reported an overall higher mortality rate in females, this became similar to males during the last few years. A national multicentre study [18] confirmed similar 30-day mortality and postoperative complication rates between genders, despite differences in clinical presentation and surgical strategies. Chemtob *et al.* [15] reported equivalent results, showing a 17% 30-day mortality in both genders, consistent with our rates. More recently, Zhang *et al.* [21] confirmed similar postoperative mortality and morbidity between males and females who underwent TAAD surgery between 2015 and 2021.

The present study also examined long-term outcome in males and females undergoing TAAD surgery. Again, no differences in 10-year survival or cumulative incidence of aortic reoperation were observed. The latest study from the IRAD registry [14] estimated 82.6% and 85.9% 5-year survival in males and females, respectively (*P* = 0.14), as well as similar freedom from aortic reintervention (87.8% in women vs 87.6% in men; *P* = 0.19). Zhou *et al.* [22] reported 5-year cumulative survival and incidence of aortic reintervention among female patients, which was not statistically different from those observed in male patients before and after matching. However, they reported only mid-term outcomes. To the best of our knowledge, only 2 studies reported on long-term results [22, 23]. Both studies demonstrated comparable cumulative survival between males and females, ranging from 50% to 60% after 10 years from surgery. In the present study, 10-year survival of males was 47.8% and that of females was 47.1% (*P* = 0.679). Nevertheless, both studies are limited by their small sample sizes. Furthermore, survival is affected by country-related life expectancy. By contrast, the present analysis included data from 3902 patients operated over a 16-year period, thus representing one of the largest European registries investigating gender-related differences in TAAD surgery.

Herein, we observed that 10-year relative survival of patients who underwent surgery for acute TAAD compared to country-, year-, age- and sex-matched general population was higher among males (0.65) compared to females (0.58). Supplementary Material, Fig. S5 demonstrated that after a drop related to early postoperative mortality occurring during the first postoperative year, relative survival slightly decreased over time, with females having had a lower observed survival than the expected one as compared to males. The drop in relative survival among females seemed to occur after 6 years after surgery and the smoothed hazard estimates increased among females at the same time interval. We hypothesize that this finding might be related to differences in late aortic-related events between genders. However, the cumulative incidences of aortic reoperation were comparable between males and females, but we do not have data on aortic degeneration which were treated conservatively because of the advanced age and high operative risk of the patient.

The time-period subanalysis revealed trends in surgical technique across years, especially in terms of more extensive aortic arch treatment and more frequent use of antegrade cerebral perfusion in both genders. However, femoral artery cannulation remained the preferred location for CBP in females, as well as supracoronary ascending aortic replacement as compared to males. Indeed, aortic root replacement had a declining trend over the years in the male cohort as well. The debate on the optimal extent of aortic resection in patients with acute TAAD began 3 decades ago, with pioneers of aortic surgery recognizing the potential importance of aortic arch replacement to reduce the risk of late aortic-related complications. As methods of cerebral protection have advanced, there has been a significant increase in the frequency of repair of the aortic arch for TAAD. The introduction of hybrid prostheses has also led to a more aggressive approach to aortic resection, potentially explaining the increase in in distal aortic surgery in both genders. On the other hand, there is ongoing controversy surrounding the treatment of the aortic root in TAAD. Studies have yielded conflicting results on the impact of root replacement on mortality, with some showing no significant effects. Recently, Lau *et al.* [24] suggested that extensive repair including root replacement was a predictor of later reoperation as compared to the conservative approach that comprised of a root-sparing. This may contribute to the observed decrease in root surgery in our study cohorts. In analysing gender differences in TAAD surgery, our study found that females tended towards a more conservative approach in both distal and proximal extension during TAAD surgery. This trend has been noted by previous researchers, such as Nappi *et al.* [23], who reported a gradual decrease in the proportion of aortic root procedures over time in men, while the proportion of aortic arch surgeries increased. Rylski *et al*. [8] alongside Huckaby*et al.* [14] reported on similar operative strategy of total arch replacement procedure in women (13.5% vs 15.2%). Finally, our data demonstrated no differences in postoperative mortality among males and females over time, as well as for major postoperative complications, except for stroke, whose incidence gradually increased over the years in all patients. It is worth noting that the frozen elephant trunk procedure was identified as an independent risk factor for stroke in males. Indeed, it is well established that duration of hypothermic circulatory arrest affects neurological outcome in aortic arch surgery [25], potentially explaining the results of our multivariate analysis. Noteworthy, no intraoperative factors emerged as predictive for stroke in females, suggesting existence of other risk factors, irrespective of the surgical approach. It has been clearly shown that females face a disproportionate burden of stroke-related mortality and disability [26, 27]. Females have substantial differences compared to males in the prevalence of diabetes, hypertension, body mass index and atrial fibrillation, but also experience some female-specific risk factors, such as endogenous levels of age-varying sex hormones [27]. Moreover, the lifetime risk of stroke is higher for females than for males, with a 1 in 4 risk of stroke for females above age 25 years, which might be a potentially crucial factor in an aged population of females experiencing TAAD [25–28]. Finally, several genetic and epigenetic influences have been explored, suggesting a crucial role of female sex in stroke pathophysiology [25].

### Limitations

The primary limitation of this study stems from its retrospective nature. However, TAAD requires urgent or emergent surgery, which explains the absence in current literature of prospective randomized studies on this highly lethal condition. Due to the retrospective nature of the study, certain specific data such as aortic diameters were not included in the analysis due to a high percentage of missing data (>30%). The study exclusively included all patients who underwent TAAD surgery. Consequently, it does not involve individuals who did not reach the hospital or those in whom surgery was deemed futile because of preoperative moribund state. Nevertheless, the objective of the study was to investigate gender differences in patients undergoing surgery, aiming to deeper understand potential differences between males and females in surgical techniques and outcomes of TAAD surgery over the last decades. Another limitation stems in the non-exchangeable characteristics between females and males given by things intrinsic to the biological state; therefore, some bias could remain also after adjustment. Finally, we acknowledge that only 1 of 29 covariates is not perfect balance (SD 0.16), but from a clinical standpoint, the preoperative antiplatelet exposure was not considered crucial for 10-year survival and cumulative incidence reoperations, which is the primary objective of this study.

## CONCLUSION

The ongoing advancements in surgical techniques and TAAD management seem to have mitigated the gender disparity reported in past decades in terms of early and late outcomes. Some differences still exist in the extent of surgery and surgical techniques between males and females, but these did not impact either early or late postoperative outcomes. However, late relative survival was still in favour of males.

## Supplementary Material

ezae242_Supplementary_Data

## Data Availability

All relevant data are within the manuscript and its supporting information files.

## Notes

Presented at the 37th EACTS Annual Meeting, Vienna, Austria, October 2023.

## ABBREVIATIONS

CI: Confidence interval
CPB: Cardiopulmonary bypass
GERAADA: German Registry for Acute Aortic Dissection Type A
HR: Hazard ratio
IQR: Interquartile range
IRAD: International Registry of Acute Aortic Dissection
NORCAAD: Nordic Consortium for Acute Type A Aortic Dissection
SHR: Subdistributional hazard ratio
STROBE: Strengthening the Reporting of Observational Studies in Epidemiology
TAAD: Type A aortic dissection
