# Supplementary material for: Role of gender in short- and long-term outcomes after surgery for type A aortic dissection: analysis of a multicentre European registry
Source: Eur J Cardiothorac Surg. 2024 Jun 26;66(1):ezae242. doi: 10.1093/ejcts/ezae242 (PMC12017467; doi:10.1093/ejcts/ezae242)
Supplement: ezae242_Supplementary_Data [file ezae242_supplementary_data.docx]

Supplementary material

**Role of Gender in Short and Long-term Outcomes after Surgery for Type A Aortic Dissection: Analysis of a Multicentre European Registry**

F. Onorati et al.

**Supplementary figure 1.** Kaplan-Meier’s estimates of survival of males and females in the overall series.

**Supplementary figure 2.** Cumulative incidences of proximal (A) and distal (B) aortic reoperations in males and females in the overall series.

**Supplementary figure 3.** Overlaying graphs of males and females distributions before and after propensity score matching.

**Supplementary figure 4.** Smoothed hazard estimates of mortality of propensity score matched pairs of males and females.

**Supplementary figure 5.** Relative survival of males and females compared to country, year, age and sex-matched general population.

**Supplementary table 1.** Overall population: baseline patient characteristics.

**Supplementary table 2.** Overall population: intraoperative findings and operative data.

**Supplementary table 3.** Overall population: early postoperative outcomes.

**Supplementary table 4.** Total arch replacement and presence of tear in the aortic arch.

**Supplementary table 5.** Time-period 2005-2009: baseline characteristics of propensity score matched patients.

**Supplementary table 6.** Time-period 2010-2014: baseline characteristics of propensity score matched patients.

**Supplementary table 7.** Time-period 2015-2021: baseline characteristics of propensity score matched patients.

**Supplementary table 8.** Time-period sub-analysis: differences in surgical approach between males and females in different study periods.

**Supplementary table 9.** Independent predictors of stroke in males in multivariable analysis.

**Supplementary table 10.** Independent predictors of stroke in females in multivariable analysis.

**
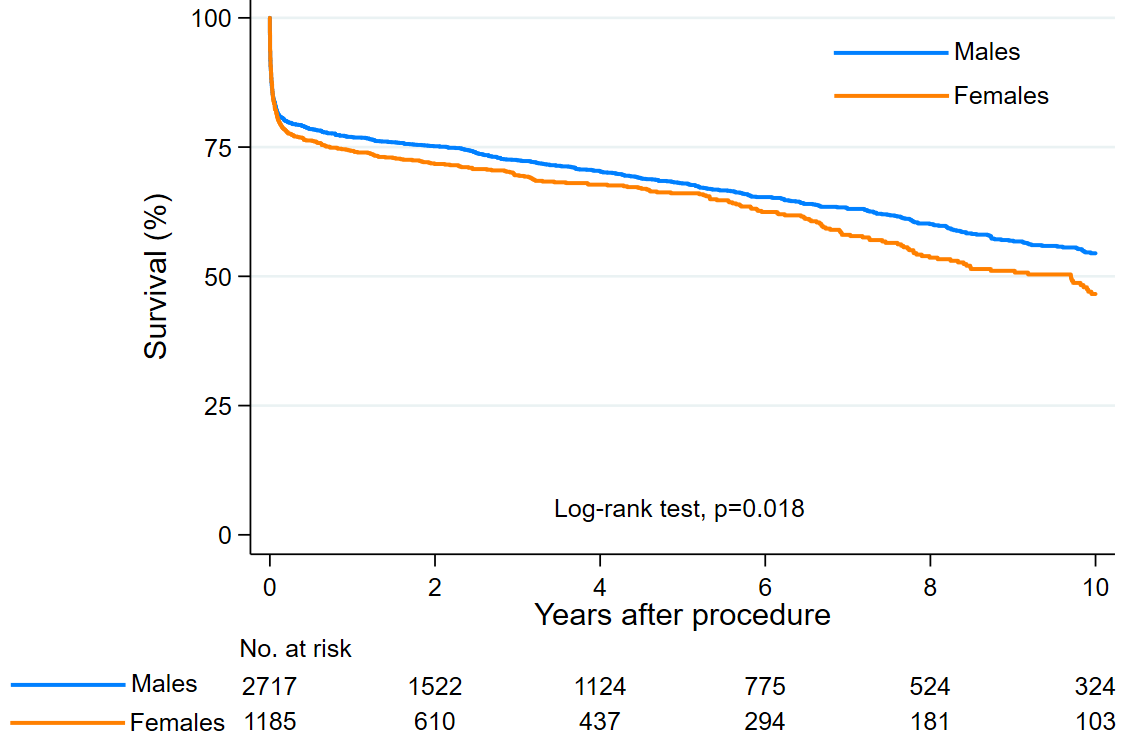
**

**Supplementary figure 1.** Kaplan-Meier’s estimates of survival of males and females in the overall series.

**
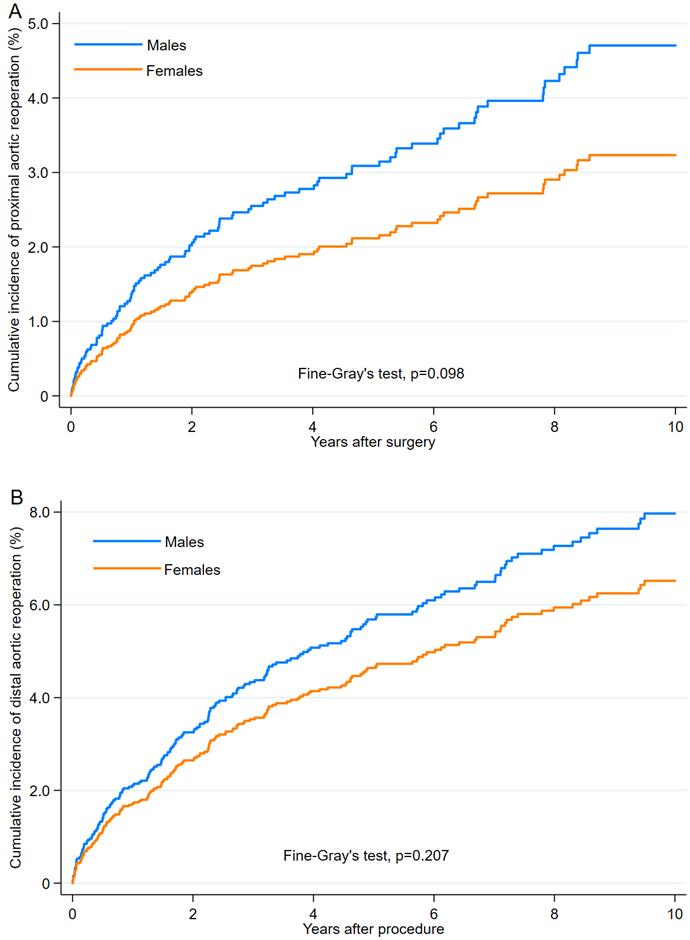
**

**Supplementary figure 2.** Cumulative incidences of proximal (A) and distal (B) aortic reoperations in males and females in the overall series.

**
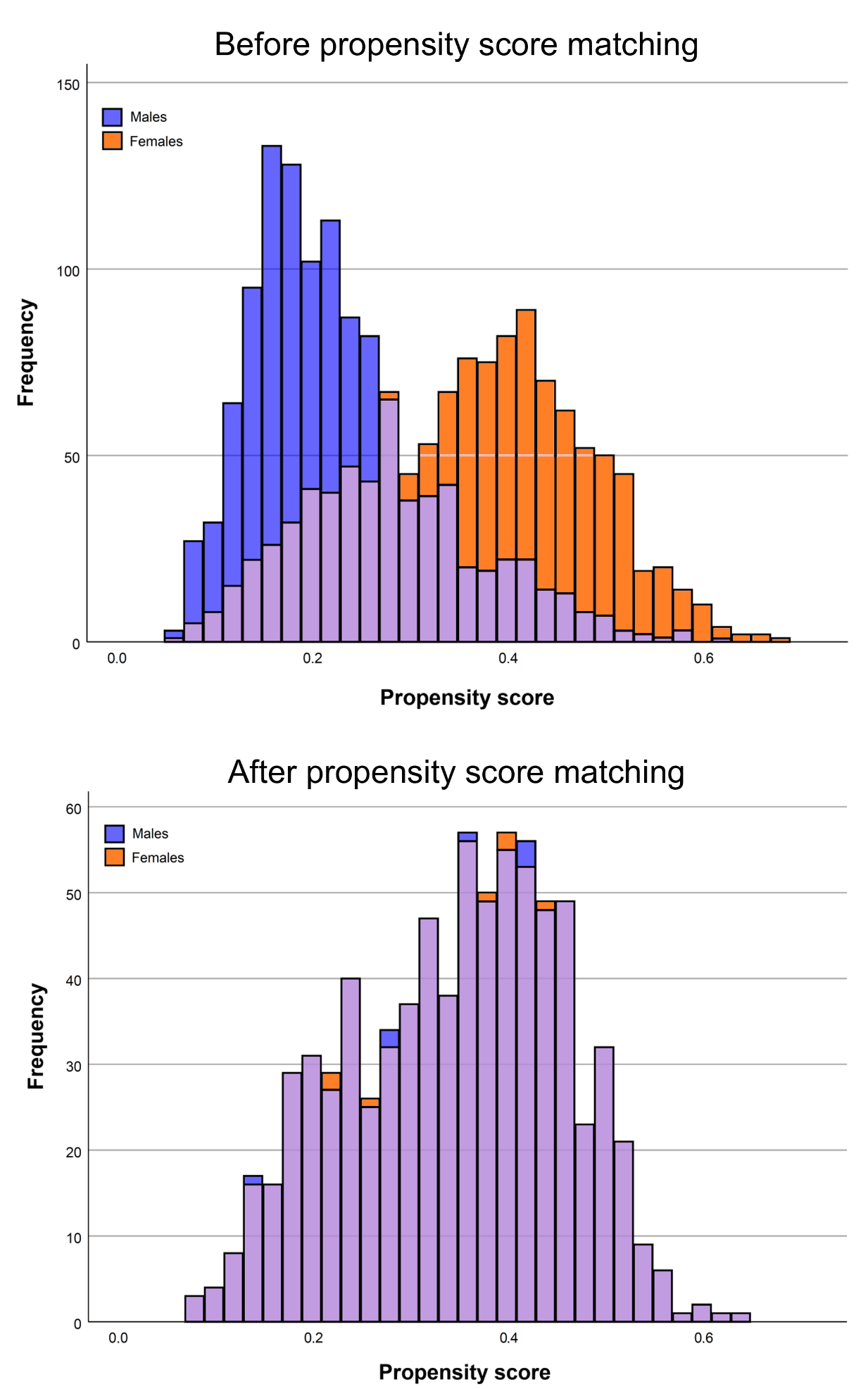
**

**Supplementary figure 3.** Overlaying graphs of males and females distributions before and after propensity score matching.

**
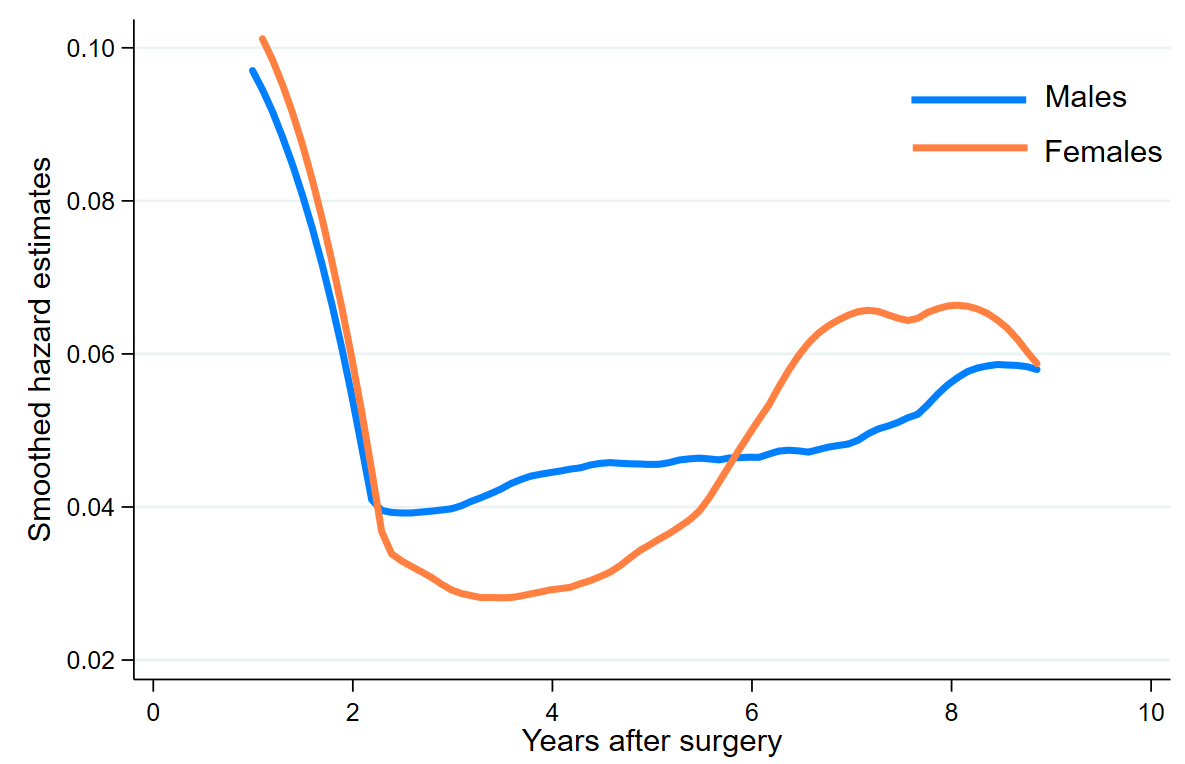
**

**Supplementary figure 4.** Smoothed hazard estimates of mortality of propensity score matched pairs of males and females.

**
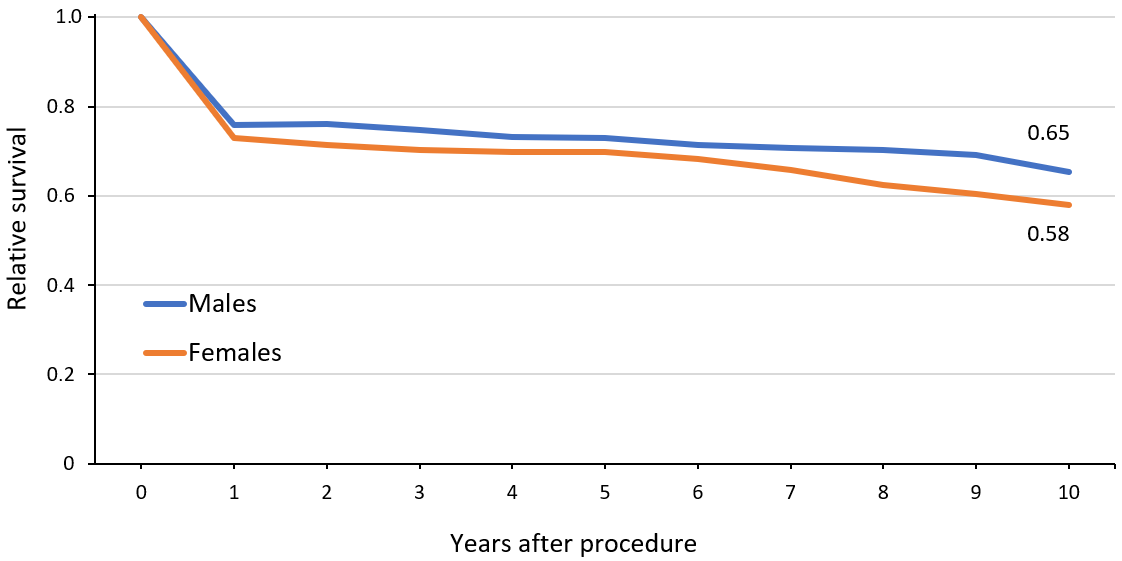
**

**Supplementary figure 5.** Relative survival of males and females compared to country, year, age and sex-matched general population.

**Supplementary table 1.** Overall series: baseline patient characteristics.

| Variables | Males  No. 2717 | Females  No. 1185 | p-value |
| --- | --- | --- | --- |
| Age, years | 61.3 (12.8) | 68.1 (12.2) | <0.001 |
| Genetic syndromes | 50 (1.8) | 31 (2.6) | 0.118 |
| Marfan syndrome | 47 (1.7) | 26 (2.2) | 0.32 |
| Loyes-Dietz syndrome | 3 (0.1) | 3 (0.3) | 0.29 |
| Ehlers-Danlos syndrome | 0 | 2 (0.2) | 0.09 |
| Family history of aortic aneurysm | 125 (4.6) | 57 (4.8) | 0.74 |
| Family history of aortic dissection | 83 (3.1) | 36 (3) | 0.97 |
| Aortitis | 9 (0.3) | 6 (0.5) | 0.42 |
| Prior cardiac surgery | 85 (3.1) | 37 (3.1) | 0.99 |
| Iatrogenic TAAD | 56 (2.1) | 47 (4) | <0.001 |
| Hypertension | 1908 (70.2) | 878 (74.1) | 0.02 |
| Diabetes | 125 (4.6) | 71 (6) | 0.07 |
| Stroke | 104 (3.8) | 49 (4.1) | 0.65 |
| Pulmonary disease | 203 (7.5) | 124 (10.5) | 0.002 |
| Extracardiac arteriopathy | 127 (4.7) | 72 (6.1) | 0.07 |
| Moderate-to-severe frailty (CFS grades 6-9) | 24 (0.9) | 23 (1.9) | 0.005 |
| Exposure to antiplatelet drugs* | 178 (6.6) | 64 (5.4) | 0.17 |
| Exposure to oral anticoagulant | 168 (6.2) | 92 (7.8) | 0.07 |
| Penn classification |  |  | 0.13 |
| a | 1545 (56.9) | 708 (59.7) |  |
| b | 698 (25.7) | 265 (22.4) |  |
| c | 177 (6.5) | 86 (7.3) |  |
| b+c | 297 (10.9) | 126 (10.6) |  |
| Cardiac massage | 125 84.6) | 42(3.5) | 0.13 |
| Invasive mechanical ventilation | 243 (8.9) | 112 (9.5) | 0.61 |
| Acute renal failure | 80 (2.9) | 35 (3) | 0.98 |
| Cardiogenic shock requiring inotropes | 445 (16.4) | 203 (17.1) | 0.56 |
| Cerebral malperfusion | 570 (21) | 260 (21.9) | 0.50 |
| Spinal malperfusion | 61 (2.2) | 21 (1.8) | 0.34 |
| Renal malperfusion | 270 (9.9) | 94 (7.9) | 0.048 |
| Mesenteric malperfusion | 122 (4.5) | 40 (3.4) | 0.11 |
| Peripheral malperfusion | 404 (14.9) | 139 (11.7) | 0.009 |
| Arterial lactate, mmol/L | 2.4 (2.3) | 2.2 (2.1) | 0.007 |

Continuous variables are reported as mean and standard deviation (in parentheses). Categorical variables are reported as counts and percentages (in parentheses). CFS=Clinical Frailty Scale; TAAD=type A aortic dissection. *: clopidogrel, ticagrelor, ticlopidine, prasugrel.

**Supplementary table 2.** Overall series: intraoperative findings and operative data.

| Variables | Males  No. 2717 | Females  No. 1185 | p-value |
| --- | --- | --- | --- |
| *Introperative findings* |  |  |  |
| Site of aortic dissection tear |  |  |  |
| Aortic root | 507 (18.7) | 183 (15.4) | 0.015 |
| Ascending aorta | 1709 (62.9) | 785 (66.2) | 0.045 |
| Aortic arch | 480 (17.7) | 166 (14) | 0.005 |
| Dissection involving sinuses of Valsava |  |  |  |
| Non-coronary sinus | 585 (21.5) | 225 (19) | 0.07 |
| Right coronary sinus | 496 (18.3) | 193 (16.3) | 0.14 |
| Left coronary sinus | 295 (10.9) | 96 (8.1) | 0.009 |
| Bicuspic aortic valve | 126 (4.6) | 25 (2.1) | <0.001 |
| *Operative data* |  |  |  |
| Arterial cannulation site |  |  |  |
| Ascending aorta/aortic arch | 436 (16.1) | 261 (22) | <0.001 |
| Innominate artery | 285 (10.5) | 105 (8.9) | 0.12 |
| Right subclavian/axillary artery | 1100 (40.5) | 422 (35.6) | 0.004 |
| Common femoral artery | 866 (31.9) | 391 (33) | 0.49 |
| Proximal aortic repair |  |  |  |
| Supracoronary aortic replacement | 1849 (68.1) | 956 (80.7) | <0.001 |
| Aortic root replacement | 868 (31.9) | 229 (19.3) | <0.001 |
| Aortic valve replacement | 139 (5.1) | 64 (1.6) | 0.71 |
| Aortic arch repair |  |  |  |
| Hemiarch repair | 1170 (43.1) | 554 (46.8) | 0.03 |
| Total arch repair | 448 (16.5) | 122 (10.3) | <.001 |
| Frozen elephant trunk procedure | 186 (6.8) | 53 (4.5) | 0.004 |
| Conventional elephant trunk procedure | 87 (3.2) | 17 (0.4) | 0.002 |
| Distal ascending aorta anastomosis | 750 (27.6) | 345 (29.1) | 0.33 |
| TEVAR during the index hospitalization | 25 (0.9) | 3 (0.1) | 0.02 |
| Cerebral perfusion strategy |  |  |  |
| Antegrade | 1819 (66.9) | 762 (64.3) | 0.11 |
| Retrograde | 116 (8.2) | 44 (6.7) | 0.54 |
| Myocardial ischemic time, min | 124.9 (60.3) | 109.05 (54.4) | <0.001 |
| CPB time, min | 223. 6 (90.3) | 198.9 (84.6) | <0.001 |

Continuous variables are reported as mean and standard deviation (in parentheses). Categorical variables are reported as counts and percentages (in parentheses). CPB=cardiopulmonary bypass time; TEVAR=Thoracic endovascular aortic repair.

**Supplementary table 3.** Overall series: early postoperative outcomes.

| Outcomes | Males  No. 2717 | Females  No. 1185 | p-value |
| --- | --- | --- | --- |
| In-hospital mortality | 480 (17.7) | 209 (17.6) | 0.98 |
| Any neurological complication | 574 (21.1) | 260 (21.9) | 0.58 |
| Stroke | 405 (14.9) | 187 (15.8) | 0.5 |
| Paraplegia /paraparesis | 141 (5.2) | 63 (5.3) | 0.87 |
| Tetraplegia | 3 (0.1) | 0 | 0.34 |
| Sepsis | 351 (12.9) | 123 (10.4) | 0.03 |
| Dialysis | 428 (15.8) | 131(11.1) | <0.001 |
| Laryngeal nerve palsy | 52 (1.9) | 19 (1.6) | 0.5 |
| Reoperation for intrathoracic bleeding | 414 (15.2) | 135 (11.4) | 0.001 |
| Deep sternal wound infection | 64 (2.4) | 25 (2.1) | 0.63 |
| Tracheostomy | 216 (7.9) | 103 (8.7) | 0.44 |
| Heart failure | 381 (14) | 171 (14.4) | 0.74 |
| IABP support | 21 (0.8) | 14 (1.2) | 0.21 |
| ECMO support | 82 (3) | 29 (2.4) | 0.34 |
| Mesenteric ischemia | 107 (2.7) | 42 (3.5) | 0.55 |
| Acute upper limb ischemia | 8 (0.3) | 5 (0.4) | 0.55 |
| Acute lower limb ischemia | 102 (3.8) | 22 (1.9) | 0.002 |
| Additional procedures for ischemic complications |  |  |  |
| Major lower limb amputation | 15 (0.6) | 2 (0.2) | 0.12 |
| Revascularization procedure for upper limb ischemia | 5 (0.2) | 3(0.3) | 0.7 |
| Revascularization procedure for lower limb ischemia | 43 (1.6) | 9 (0.8) | 0.04 |
| Revascularization for mesenteric ischemia | 6 (0.2) | 1 (0.1) | 0.68 |
| Revascularization for renal ischemia | 7 (0.3) | 0 | 0.11 |
| Surgery for intestinal complication | 17 (0.6) | 1 (0.1) | 0.02 |
| Aortic fenestration | 6 (0.2) | 0 | 0.1 |
| ICU stay, days | 9.7 (13.6) | 9.2 (11.6) | 0.26 |

Continuous variables are reported as mean and standard deviation (in parentheses). Categorical variables are reported as counts and percentages (in parentheses). ECMO=extracorporeal membrane oxygenation; IABP=intra-aortic balloon pump; ICU=intensive care unit.

**Supplementary table 4.** Overall series: total arch replacement and presence of tear in the aortic arch.

|  | Total arch repair  No. 193 | Males  No. 112 | Females  No. 81 | p-value |
| --- | --- | --- | --- | --- |
| No tear in the aortic arch | 105 (54.4) | 60 (53.6) | 45 (55.6) | 0.78 |
| Tear in the aortic arch | 88 (45.6) | 52 (46.4) | 36 (44.4) |  |

Categorical variables are reported as counts and percentages (in parentheses).

**Supplementary table 5.** Time-period 2005-2009: baseline characteristics of propensity score matched patients.

| Variables | Males  No. 124 | Females  No. 124 | p-value | SD |
| --- | --- | --- | --- | --- |
| Age, years | 64.8 (11.1) | 64.8 (10.9) | 0.99 | 0 |
| Genetic syndromes | 1 (0.8) | 1 (0.8) | 1 | 0 |
| Prior cardiac surgery | 6 (4.8) | 4 (1.6) | 0.75 | 0.1 |
| Iatrogenic TAAD | 6 (4.8) | 5 (4.0) | 0.76 | 0.03 |
| Hypertension | 93 (75.0) | 95 (76.6) | 0.77 | 0.02 |
| Diabetes | 6 (4.8) | 5 (4.0) | 0.76 | 0.03 |
| Prior stroke | 3 (2.4) | 6 (4.8) | 0.31 | 0.1 |
| Pulmonary disease | 12 (9.7) | 12 (9.7) | 1 | 0 |
| Extracardiac arteriopathy | 9 (7.3) | 11 (8.9) | 0.64 | 0.05 |
| Moderate-to-severe frailty (CFS grade 6-9) | 1 (0.8) | 1 (0.8) | 1 | 0 |
| Penn classification |  |  | 0.53 | 0.08 |
| a | 79 (63.7) | 86 (69.4) |  |  |
| b | 22 (17.7) | 23 (18.5) |  |  |
| c | 9 (7.3) | 7 (5.6) |  |  |
| b+c | 14 (11.3) | 8 (6.5) |  |  |
| Cardiac massage | 7 (5.6) | 5 (4.0) | 0.55 | 0.16 |
| Invasive mechanical ventilation | 9 (7.3) | 9 (7.3) | 1 | 0 |
| Acute renal impairment | 7 (5.6) | 3 (2.4) | 0.33 | 0.16 |
| Cardiogenic shock requiring inotropes | 22 (17.7) | 15 (12.1) | 0.21 | 0.1 |
| Cerebral malperfusion | 21 (17.7) | 22 (17.7) | 0.87 | 0 |
| Spinal malperfusion | 2 (1.6) | 1 (0.8) | 0.56 | 0.07 |
| Renal malperfusion | 11 (8.9) | 7 (5.6) | 0.33 | 0.1 |
| Mesenteric malperfusion | 5 (5.0) | 1 (0.8) | 0.09 | 0.2 |
| Peripheral malperfusion | 15 (12.1) | 12 (9.7) | 0.54 | 0.07 |
| Arterial lactate, mmol/L | 1.9 (1.3) | 2.2 (1.7) | 0.31 | 0.1 |

Continuous variables are reported as mean and standard deviation (in parentheses). Categorical variables are reported as counts and percentages (in parentheses). CFS=Clinical Frailty Scale; SD=standardized difference; TAAD=type A aortic dissection.

**Supplementary table 6.** Time-period 2010-2014: baseline characteristics of propensity score matched patients.

| Variables | Males  No. 217 | Females  No. 217 | p-value | SD |
| --- | --- | --- | --- | --- |
| Age, years | 65.2 (12.4) | 64.4 (12.9) | 0.5 | 0.06 |
| Genetic syndromes | 6 (2.8) | 8 (3.7) | 0.6 | 0.05 |
| Prior cardiac surgery | 14 (6.5) | 10 (4.6) | 0.4 | 0.08 |
| Iatrogenic TAAD | 8 (3.7) | 5 (2.3) | 0.4 | 0.08 |
| Hypertension | 159 (73.3) | 153 (70.5) | 0.5 | 0.06 |
| Diabetes | 11 (5.1) | 14 (6.5) | 0.5 | 0.06 |
| Prior stroke | 14 (6.5) | 6 (2.8) | 0.07 | 0.2 |
| Pulmonary disease | 24 (11.1) | 24 (11.1) | 1 | 0 |
| Extracardiac arteriopathy | 11 (5.1) | 9 (4.1) | 0.6 | 0.04 |
| Moderate-to-severe frailty (CFS grade6-9) | 3 (1.4) | 3 (1.4) | 1 | 0 |
| Penn classification |  |  | 0.9 | 0.02 |
| a | 126 (58.1) | 130 (59.9) |  |  |
| b | 54 (24.9) | 49 (22.6) |  |  |
| c | 10 (4.6) | 9 (4.1) |  |  |
| b+c | 27 (12.4) | 29 (13.4) |  |  |
| Cardiac massage | 7 (3.2) | 9 (4.1) | 0.6 | 0.05 |
| Invasive mechanical ventilation | 25 (11.5) | 24 (11.1) | 0.9 | 0.01 |
| Acute renal impairment | 5 (2.3) | 8 (3.7) | 0.4 | 0.08 |
| Cardiogenic shock requiring inotropes | 37 (17.1) | 35 (16.1) | 0.8 | 0.02 |
| Cerebral malperfusion | 53 (24.4) | 45 (20.7) | 0.4 | 0.08 |
| Spinal malperfusion | 1 (0.5) | 4 (1.8) | 0.2 | 0.1 |
| Renal malperfusion | 14 (6.5) | 17 (7.8) | 0.6 | 0.05 |
| Mesenteric malperfusion | 6 (2.8) | 9 (4.1) | 0.4 | 0.07 |
| Peripheral malperfusion | 37 (17.1) | 37 (17.1) | 1 | 0 |
| Arterial lactate, mmol/L | 2.2(2.1) | 2.2 (2.1) | 0.8 | 0 |

Continuous variables are reported as mean and standard deviation (in parentheses). Categorical variables are reported as counts and percentages (in parentheses). CFS=Clinical Frailty Scale; SD=standardized difference; TAAD=type A aortic dissection.

**Supplementary table 7.** Time-period 2015-2021: baseline characteristics of propensity score matched patients.

| Variables | Males  No. 468 | Females  No. 468 | p-value | SD |
| --- | --- | --- | --- | --- |
| Age, years | 66.9 (11.8) | 66.8 (12) | 0.3 | 0.008 |
| Genetic syndromes | 8 (1.7) | 6 (1.3) | 0.6 | 0.03 |
| Prior cardiac surgery | 11 (2.4) | 10 (2.1) | 0.8 | 0.02 |
| Iatrogenic TAAD | 12 (2.6) | 9 (1.9) | 0.5 | 0.04 |
| Hypertension | 339 (72.4) | 350 (74.8) | 0.41 | 0.05 |
| Diabetes | 23 (4.9) | 26 (5.6) | 0.6 | 0.03 |
| Prior stroke | 24 (5.1) | 23 (4.9) | 0.9 | 0.009 |
| Pulmonary disease | 44 (9.4) | 40 (8.5) | 0.6 | 0.03 |
| Extracardiac arteriopathy | 29 (6.2) | 28 (6.0) | 0.9 | 0.008 |
| Moderate-to-severe frailty (CFS grade6-9) | 6 (1.3) | 7 (1.5) | 0.8 | 0.01 |
| Penn clssification |  |  | 0.82 | 0.01 |
| a | 276 (59) | 267 (57.1) |  |  |
| b | 116 (24.8) | 116 (24.8) |  |  |
| c | 38 (8.1) | 39 (8.3) |  |  |
| b+c | 38 (8.1) | 46 (9.8) |  |  |
| Cardiac massage | 20 (4.3) | 21 (4.5) | 0.8 | 0.008 |
| Invasive mechanical ventilation | 44 (9.4) | 41 (8.8) | 0.7 | 0.02 |
| Acute renal impairment | 8 (1.7) | 16 (3.4) | 0.09 | 0.1 |
| Cardiogenic shock requiring inotropes | 69 (14.7) | 80 (17.1) | 0.4 | 0.06 |
| Cerebral malperfusion | 93 (19.9) | 105 (22.4) | 0.3 | 0.06 |
| Spinal malperfusion | 10 (2.1) | 7 (1.5) | 0.5 | 0.04 |
| Renal malperfusion | 53 (11.3) | 53 (11.3) | 1 | 0 |
| Mesenteric malperfusion | 19 (4.1) | 19 (4.1) | 1 | 0 |
| Peripheral malperfusion | 52 (11.1) | 51 (10.9) | 0.9 | 0.006 |
| Arterial lactate, mmol/L | 2.4 (2.3) | 2.3 (2.1) | 0.4 | 0.01 |

Continuous variables are reported as mean and standard deviation (in parentheses). Categorical variables are reported as counts and percentages (in parentheses). CFS=Clinical Frailty Scale; SD=standardized difference; TAAD=type A aortic dissection.

**Supplementary table 8.** Time-period sub-analysis: differences in surgical approach between males and females in different study periods.

|  | 2005-2009  124 pairs | 2010-2014  217 pairs | 2015-2021  468 pairs | p-value |
| --- | --- | --- | --- | --- |
|  | **Supracoronary replacement** | | |  |
| Males, No. (%) | 82 (66.1) | 153 (70.5) | 353 (75.4) | *0.08* |
| Females, No. (%) | 89 (71.8) | 172 (79.3) | 386 (82.5) | *0.03* |
| *p-value* | *0.34* | *0.03* | *0.008* |  |
|  | **Aortic Root Surgery** | | |  |
| Males, No. (%) | 42 (33.9) | 64 (29.5) | 115 (24.6) | *0.08* |
| Females, No. (%) | 35 (28.2) | 45 (20.7) | 82 (17.5) | *0.008* |
| *p-value* | *0.33* | *0.035* | *0.008* |  |
|  | **Hemiarch repair** | | |  |
| Males, No. (%) | 57 (23) | 112 (51.6) | 205 (43.8) | *0.16* |
| Females, No. (%) | 51 (20.6) | 98 (45.2) | 221 (47.2) | *0.47* |
| *p-value* | *0.44* | *0.18* | *0.29* |  |
|  | **Total aortic arch surgery** | | |  |
| Males, No. (%) | 12 (9.7) | 28 (12.9) | 77 (16.5) | *0.12* |
| Females, No. (%) | 9 (7.3) | 12 (5.5) | 72 (15.4) | *<0.001* |
| *p-value* | *0.49* | *0.008* | *0.65* |  |
|  | **Frozen elephant trunk** | | |  |
| Males, No. (%) | 0 | 5 (2.3) | 49 (10.5) | *<0.001* |
| Females, No. (%) | 0 | 2 (0.9) | 41 (8.8) | *<0.001* |
| *p-value* | *0* | *0.25* | *0.37* |  |
|  | **Distal ascending aorta anastomosis** | | |  |
| Males, No. (%) | 41 (33.1) | 56 (25.8) | 116 (24.8) | *0.17* |
| Females, No. (%) | 47 (37.9) | 68 (31.3) | 116 (24.8) | *0.009* |
| *p-value* | *0.43* | *0.20* | *1.0* |  |
|  | **TEVAR during the index hospitalization** | | |  |
| Males, No. (%) | 1 (0.8) | 0 | 1 (0.2) | *0.34* |
| Females, No. (%) | 0 | 0 | 2 (0.4) | *0.48* |
| *p-value* | *0.32* | - | 0.50 |  |
|  | **Axillary artery cannulation** | | |  |
| Males, No. (%) | 37 (29.8) | 94 (43.3) | 224 (47.9) | *0.002* |
| Females, No. (%) | 33 (26.6) | 83 (38.2) | 190 (40.6) | *0.017* |
| *p-value* | *0.60* | *0.28* | *0.025* |  |
|  | **Femoral artery cannulation** | | |  |
| Males, No. (%) | 54 (43.5) | 65 (30) | 99 (21.2) | *<0.001* |
| Females, No. (%) | 70 (56.5) | 82 (37.8) | 122 (26.1) | *0<.001* |
| *p-value* | *0.042* | *0.08* | *0.07* |  |
|  | **Antegrade cerebral perfusion** | | |  |
| Males, No. (%) | 68 (54.8) | 158 (72.8) | 331(70.7) | *<0.001* |
| Females, No. (%) | 59 (47.6) | 132 (60.8) | 328 (70.1) | *<0.001* |
| *p-value* | *0.25* | *0.008* | *0.83* |  |

Categorical variables are reported as counts and percentages (in parentheses). TEVAR=thoracic endovascular aortic repair.

**Supplementary table 9.** Independent predictors of stroke in males in multivariable analysis.

| Variables | Odds ratio | 95% CI | p-value |
| --- | --- | --- | --- |
| Exposure to potent antiplatelet drugs | 1.54 | 1.03 - 2.31 | 0.036 |
| History of prior stroke | 2.32 | 1.42 - 3.79 | <0.001 |
| Cerebral malperfusion | 2.10 | 1.66 - 2.73 | <0.001 |
| Frozen elephant trunk | 1.54 | 1.02 - 2.32 | 0.042 |
| CPB time | 1.001 | 1.00 -1.003 | 0.028 |

CI=confidence interval; CPB=cardiopulmonary bypass.

**Supplementary table 10.** Independent predictors of stroke in females in multivariable analysis.

|  | Multivariate analysis | | |
| --- | --- | --- | --- |
| Variables | **Odds ratio** | **95%CI** | **p-value** |
| Arterial lactate | 1.14 | 1.06 - 1.20 | <0.001 |
| Invasive mechanical ventilation | 1.76 | 1.05 - 2.97 | 0.033 |
| Cerebral malperfusion | 1.70 | 1.12 - 2.58 | 0.013 |

CI=confidence interval.
